# Interstitial Emphysema as a Rare Radiographic Presentation of Bronchial Dehiscence after Lung Transplant

**DOI:** 10.1155/2020/8830361

**Published:** 2020-12-26

**Authors:** Linda P. Vien, Robert M. Marron, Abbas Charlie, Maruti Kumaran, James C. Brown

**Affiliations:** ^1^Department of Medicine, Temple University Hospital, 3401 North Broad Street, Philadelphia, PA 19140, USA; ^2^Department of Thoracic Medicine and Surgery, Temple University Hospital, 3401 North Broad Street, Philadelphia, PA 19140, USA; ^3^Department of Radiology, Temple University Hospital, 3401 North Broad Street, Philadelphia, PA 19140, USA

## Abstract

Airway complications after lung transplantation are a major cause of morbidity and mortality. Bronchial dehiscence presents within a month of lung transplantation and is typically diagnosed radiographically as a sentinel gas pocket at the anastomotic site and confirmed with bronchoscopy. A 66-year-old man with idiopathic pulmonary fibrosis who underwent a right lung transplantation 4 weeks prior developed chest pain with palpable crepitus over his right chest wall. A chest X-ray revealed subcutaneous emphysema and a small right-sided pneumothorax. Computed tomography (CT) of the thorax without contrast revealed a gas pocket at the anastomotic site in the mediastinum as well as interstitial emphysema around the proximal bronchi of the right lung that had worsened when compared to CT from 11 days prior. A review of prior CT demonstrated interstitial emphysema without evidence of a sentinel gas pocket. These findings suggest that interstitial emphysema was the initial radiographic manifestation of the bronchial anastomotic site dehiscence. Interstitial emphysema is typically self-limiting, but severe cases can lead to major complications. Interstitial emphysema outside of the immediate postoperative period should be recognized as a possible early radiographic sign of bronchial dehiscence in lung transplant patients with vigilant monitoring of potential complications and strong consideration for early bronchoscopic investigation.

## 1. Introduction

Bronchial anastomotic site dehiscence typically occurs 2-4 weeks after lung transplantation and occurs in 1-10% of patients [1, 2]. It occurs as a result of bronchial ischemia and extreme mucosal necrosis due to disruption of native bronchial circulation and lack of systemic blood supply to the anastomotic region immediately after transplantation; reestablishment of bronchial artery circulation through collateral formation can take up to four weeks [3]. The most common radiographic finding is a sentinel gas pocket at the anastomotic site on computed tomography (CT) imaging [1, 4]. Indirect signs of bronchial anastomotic site dehiscence include a persistent pneumothorax and subcutaneous emphysema [1]. Risk factors for bronchial dehiscence include right lung anastomoses, primary graft dysfunction, microbiological contamination of the donor lung, high-dose perioperative steroids, telescoped anastomoses, inadequate organ preservation, prolonged organ ischemia times, discrepancy in size between donor and recipient bronchi, prolonged donor or recipient mechanical ventilation, and immunosuppression, most notably sirolimus [2, 3, 5, 6]. We report an atypical presentation of bronchial anastomotic site dehiscence in a recent lung transplant patient presenting with an initial radiographic finding of interstitial emphysema rather than a sentinel gas pocket.

## 2. Case Presentation

A 66-year-old male with chronic hypoxemic respiratory failure secondary to advanced interstitial pulmonary fibrosis, coronary artery disease, pulmonary hypertension, systemic lupus erythematosus, hypertension, and obesity presented for a single right lung transplantation which was performed with end-to-end anastomosis. His operation was uneventful with no significant major intraoperative complications or use of extracorporeal membrane oxygenation. He was extubated within 24 hours posttransplant after an airway and anastomosis inspection via flexible bronchoscopy was unremarkable. The patient was treated with antibiotics for methicillin-sensitive *Staphylococcus aureus* of the donor lung; other cultures were negative. The patient was to receive tacrolimus, mycophenolate, and prednisone for immunosuppression and nebulized amphotericin and intravenous valganciclovir for fungal and cytomegalovirus prophylaxis. No primary graft dysfunction was reported in the first 72 hours after surgery. CT without contrast was obtained on postoperative day 5 to evaluate a right lung basilar opacity, which showed extensive ground-glass opacities in the transplanted right lung, concerning reperfusion edema versus acute rejection. The patient was given diuretics, and glucocorticoid dosing was increased from prednisone 5 mg daily to methylprednisolone 40 mg intravenously twice a day. His postoperative course was further complicated by hypoxemia, for which a CT angiography was performed and showed a small distal pulmonary embolism in the right lower lobe, decreased ground-glass opacities in the right lung, and increased patchy basilar platelike peribronchial opacities for which rejection could not be ruled out. The patient received three days of pulse glucocorticoid dosing of methylprednisolone 250 mg IV daily, which was subsequently decreased. Persistent abnormalities on chest radiography and continued hypoxemia led to additional imaging. CT of the thorax on postoperative day 15 showed right allograft opacities, for which glucocorticoids were continued out of concern for rejection. Subsequent bronchoscopy on postoperative day 17 showed an abnormal area of eschar around the anastomotic site with a pale yellow-brown mucosa suggestive of ischemia. Cultures from the bronchoscopy were negative. A transbronchial biopsy of the right lower lobe was performed, and pathology showed organizing pneumonia without evidence of rejection. Donor-specific antibody serology did not suggest antibody-mediated rejection. Steroids were continued and tapered in an attempt to balance treating his organizing pneumonia while hoping to avoid the complications given the presence of anastomotic eschar on bronchoscopy.

**Figure 1:**
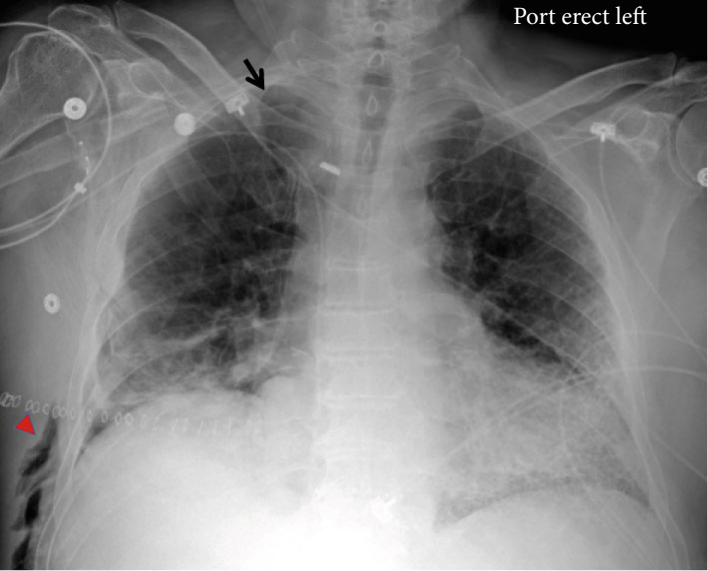
Chest X-ray showed right-sided subcutaneous emphysema (red caret) with a small right-sided pneumothorax (black arrow).

**Figure 2:**
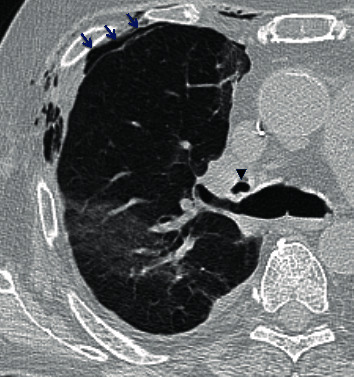
Axial chest CT showed a sentinel pocket of gas adjacent to the right bronchial anastomotic site (black caret); an anterior pneumothorax (blue arrows) is also seen.

**Figure 3:**
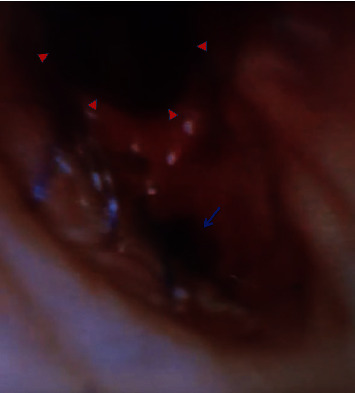
Bronchoscopy showed partial dehiscence of the right mainstem bronchus surgical anastomosis (blue arrow) directly opposite to the right upper lobe orifice (red carets) and distal to sutures.

**Figure 4:**
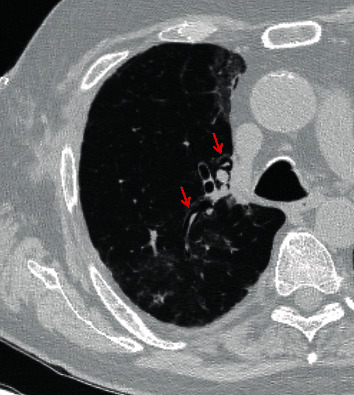
Initial axial chest CT two weeks prior shows interstitial emphysema along the bronchovascular bundle (red arrows).

**Figure 5:**
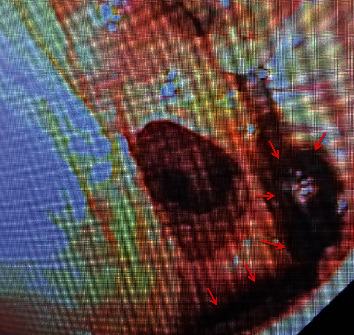
Repeat bronchoscopy 32 days posttransplantation showed right mainstem bronchial anastomotic site dehiscence (red arrows).

On postoperative day 26, the patient developed chest pain. Vital signs were as follows: temperature was 36.9 degrees Celsius, blood pressure was 108/61 mmHg, heart rate was 67 beats per minute, respiratory rate was 20 breaths per minute, and oxygen saturation was 97% on 20 liters of high-flow nasal cannula with fraction of inspired oxygen of 60%. On physical exam, the patient had rhonchi at the right base and right-sided subcutaneous emphysema on the anterior chest with no respiratory distress or accessory muscle use. A portable anterior-posterior chest X-ray showed right-sided subcutaneous emphysema with a small right-sided pneumothorax (Figure 1). CT of the thorax showed a moderate-sized right pneumothorax with spontaneous decompression into the anterior chest wall with subcutaneous emphysema, a sentinel pocket of air adjacent to the anastomotic site, and interstitial emphysema around the right lower lobe proximal bronchus, all of which were concerning bronchial dehiscence (Figure 2). The patient was transferred to the medical intensive care unit and was intubated for bronchoscopy for airway inspection. Bronchoscopy showed partial dehiscence of the right mainstem bronchus surgical anastomosis directly opposite to the right upper lobe orifice and distal to sutures with a surrounding stable hematoma and no bubbling apparent when flushed with saline (Figure 3). Retrospective evaluation of the prior CT thorax two weeks after transplantation showed air tracking along the bronchovascular bundle, suggestive of interstitial emphysema (Figure 4). Repeat bronchoscopy 32 days posttransplantation, seen in Figure 5, showed right mainstem bronchus surgical anastomotic dehiscence with the International Society for Heart and Lung Transplantation (ISHLT) grading as follows: ischemia and necrosis (extent I-a, location I-a), dehiscence (extent D-a, location D-c), stenosis (location S-a), and malacia (none). The right mainstem bronchus anastomotic dehiscence was managed conservatively with a reduction in glucocorticoid dosage, addition of prophylactic antibiotics, and serial bronchoscopy.

## 3. Discussion

Interstitial emphysema is defined as air dissecting into the interstitial space surrounding the bronchovascular bundles [7, 8]. This finding, typically seen in neonates with respiratory distress syndrome and positive pressure ventilation on mechanical ventilators, is rarely seen in adults [7, 8]. Interstitial emphysema is an atypical presentation of anastomotic site dehiscence after lung transplantation. Rare cases of interstitial emphysema have been reported after lung transplant [8, 9]. A retrospective study found that out of 17 patients with confirmed bronchial dehiscence after transplantation, there was one case of pulmonary interstitial emphysema in a patient with right bronchial dehiscence after undergoing bilateral lung transplantation [9].

Abnormal airway pressures can cause rupture of alveolar walls and subsequent transport of air from ruptured alveoli to the mediastinum via pulmonary vessels, leading to a pneumothorax and pneumomediastinum [10]. Physiologic and pathologic phenomena that are associated with interstitial emphysema are atelectasis, pneumonia, chest trauma, bronchiolitis, asthma, and most commonly a need for mechanical ventilation. Interstitial emphysema has been reported in acute respiratory distress syndrome and positive pressure ventilation where peak airway pressures exceed 30-40-centimeter H_2_O [11, 12]. Age also plays a factor. Neonates, particularly preterm babies, are at increased risk for interstitial emphysema due to reduced surfactant levels [12].

Mild interstitial emphysema is self-limiting. Severe interstitial emphysema can lead to complications including compression atelectasis, pneumomediastinum, pneumothorax, and hemodynamic effects from compression of the pulmonary vasculature [10, 12]. Management strategies include lateral decubitus positioning, inhaled helium or nitric oxide, mainstem bronchus intubation and occlusion for unilateral disease, high-frequency ventilation, extracorporeal membrane oxygenation, and lobectomy [12, 13].

Interstitial emphysema on the CT thorax 11 days prior to the patient developing symptoms may have been evidence of an atypical radiographic presentation of bronchial anastomotic site dehiscence, which was later confirmed with the classic CT finding of a sentinel gas pocket. While interstitial emphysema can occur postoperatively and is self-limiting, persistent interstitial emphysema and subcutaneous emphysema warrant closer monitoring for possible bronchial anastomotic site dehiscence. Our patient had risk factors for bronchial anastomotic site dehiscence including right-sided anastomosis, microbial contamination of the donor lung, and high glucocorticoid dosing due to initial concerns for possible rejection.

Our patient received an end-to-end anastomosis. End-to-end anastomoses are associated with a lower risk of bronchial anastomotic complications as compared to telescoped anastomoses in patients who received single lung transplantation, with bronchial dehiscence seen in 25% of telescoped bronchial anastomoses compared to 2% of end-to-end bronchial anastomoses [14]. In a telescoped anastomosis, the donor bronchus is folded inwards into the recipient bronchus with sutures performed both anteriorly and posteriorly. Telescoped anastomoses also have an increased risk of stenosis due to a narrower airway and an increased risk of infection [15]. The end-to-end single running suture has also been associated with good results with airway healing [16]. Shorter length of the donor bronchus is preferred given risk of ischemia with excessive length [15]. Prolonged donor mechanical ventilation and donor-recipient bronchial size mismatch are also associated with airway complications.

Bronchial dehiscence should be suspected in patients with subcutaneous emphysema, worsening shortness of breath, pneumothorax, and infection within the first month of lung transplantation [3]. A chest X-ray and CT of the chest are initial noninvasive imaging modalities for diagnosis. Visualization with bronchoscopy confirms diagnosis. Routine management of dehiscence includes serial bronchoscopy for airway inspection, treatment of infections, reduction in glucocorticoid dosing to facilitate healing, and stent placement for extensive necrosis [2, 3]. Surgical repair is reserved for severe cases or complete dehiscence.

## 4. Conclusion

Recognition of interstitial emphysema outside of the immediate postoperative period on a chest X-ray and/or CT imaging as a sign of early bronchial dehiscence should prompt closer monitoring with serial exams, chest imaging, and bronchoscopy. Early diagnosis and treatment can prevent further complications.

## Data Availability

Data sharing is not applicable to this article as no new data were created or analyzed.
